# The Value of Pharmaceutical Industry-Sponsored Patient Registries in Oncology Clinical Research

**DOI:** 10.1093/oncolo/oyad110

**Published:** 2023-06-07

**Authors:** E Dawn Flick, Howard R Terebelo, Susan Fish, Amani Kitali, Vrinda Mahajan, Melissa Nifenecker, Kristen Sullivan, Paul Thaler, Sarah Ussery, David L Grinblatt

**Affiliations:** Worldwide Health Economics and Outcomes Research (HEOR), Bristol Myers Squibb, San Francisco, CA, USA; Providence Cancer Institute, Southfield, MI, USA; Worldwide Health Economics and Outcomes Research (HEOR), Bristol Myers Squibb, San Francisco, CA, USA; US Medical Affairs, Bristol Myers Squibb, Summit, NJ, USA; Corporate Medical Affairs, Global Scientific Communications, Bristol Myers Squibb, Summit, NJ, USA; Research and Early Development Alliances, Bristol Myers Squibb, Summit, NJ, USA; Worldwide Health Economics and Outcomes Research (HEOR), Bristol Myers Squibb, San Francisco, CA, USA; US Medical Affairs, Bristol Myers Squibb, Summit, NJ, USA; US Medical Affairs, Bristol Myers Squibb, Summit, NJ, USA; NorthShore University HealthSystem, Evanston, IL, USA

**Keywords:** registry, oncology, observational study, prospective cohort study, real-world evidence

## Abstract

In May 2019, the US Food and Drug Administration (FDA) released the Framework for FDA’s Real-World Evidence (RWE) Program, a draft guidance to evaluate the potential use of real-world data in facilitating regulatory decisions. As a result, pharmaceutical companies and medical communities see patient registries, which are large, prospective, noninterventional cohort studies, as becoming increasingly important in providing evidence of treatment effectiveness and safety in clinical practice. Patient registries are designed to collect longitudinal clinical data on a broad population to address critical medical questions over time. With their large sample sizes and broad inclusion criteria, patient registries are often used to generate RWE in the general and underrepresented patient populations that are less likely to be studied in controlled clinical trials. Here, we describe the value of industry-sponsored patient registries in oncology/hematology settings to healthcare stakeholders, in drug development, and in fostering scientific collaboration.

## Teaser

Patient registries provide real-world evidence of treatment effectiveness and safety in clinical practice. This review discusses the characteristics and use of patient registries in oncology and hematology settings and describes the value of industry-sponsored oncology registries for healthcare stakeholders and for fostering scientific collaboration.

**Implications for Practice**

Patient registries provide real-world evidence on the safety and effectiveness of medical interventions in large, clinically diverse patient populations. This review discusses the characteristics and use of patient registries in oncology and describes the value of industry-sponsored oncology registries to healthcare stakeholders and for fostering scientific collaboration.

## Introduction

In May 2019, the US Food and Drug Administration (FDA) released the Framework for FDA’s Real-World Evidence (RWE) Program, a draft guidance to evaluate the potential use of real-world data (RWD) in facilitating regulatory decisions.^1^ The guidance was a clear indication of the increasing importance of RWE to regulatory agencies and to the medical community. As a result, pharmaceutical companies and medical communities see patient registries as increasingly important in providing reliable RWE of treatment effectiveness and safety in clinical practice.^2,3^ A broad, widely accepted definition of patient registries is “an organized system that uses observational study methods to collect uniform data (clinical and other) to evaluate specified outcomes for a population defined by a particular disease, condition, or exposure and that serves one or more stated scientific, clinical, or policy purpose(s).”^4^ The registries that will be discussed in this article are conducted by pharmaceutical companies and are defined as large, prospective, observational (noninterventional) cohort studies of populations defined by a specific disease or a specific treatment in which data are collected longitudinally in a systematic manner from real-world clinical settings.^4^ Pharmaceutical companies conduct registries, as either a postmarketing commitment to a regulatory authority or voluntarily to observe the safety and effectiveness of their products and to better understand evolving disease and treatment landscapes. This manuscript focuses on voluntary, pharmaceutical industry-sponsored patient registries in solid tumor and hematologic malignancies, most on which the authors have directly participated, and on the value of these studies to healthcare stakeholders, in drug development and in fostering scientific collaboration.

### The Value of Oncology Patient Registries to Healthcare Stakeholders and in Drug Development

Numerous stakeholders are informed by industry-sponsored oncology registries, including the medical community, patients, regulatory agencies, payors, and pharmaceutical companies. The cancer treatment landscape has changed rapidly over the last several decades, with the introduction of genomic mapping and genomic sequencing technology, significant advancements in the engineering of complex antibodies and targeted therapies, and the utilization of new combinations of novel agents with traditional therapies. Large randomized controlled trials (RCTs) are often not feasible and might not be considered ethical to conduct in rare cancer subtypes or in advanced malignancies. These disease settings may necessitate the approval of drugs based on relatively small studies, single-arm trials, or meta-analyses.^5^ In addition, registrational clinical trials conducted in a specific geographic region or country may have limited applicability in broader clinical practice due to differences in patient demographics, clinical practice patterns, and resource availability.^6^ Clinical trials are purposely designed to include more homogeneous populations and must adhere to strict study protocols to examine the causal effects of particular clinical interventions. Hence, they tend to have more stringent eligibility criteria that often exclude older, frail, or sicker patients. These criteria may skew the trial population toward a healthier population than patients in the general population.^4,7^

Compared with oncology clinical trials, patient registries have fewer barriers to participation (eg, broader inclusion criteria and limited exclusion criteria), which enables the analysis of treatment patterns, clinical outcomes, and adverse events (AEs) in more heterogeneous patient populations, particularly in specific subsets of patients who are not typically included in trials or tend to distrust trial participation, such as elderly patients, patients with multiple comorbidities, and patients from minority racial groups.^8-11^ Depending on the incidence of the disease of interest and the treatment penetration, registries often enroll large numbers (hundreds to thousands) of patients from community, academic, and government clinical practices (patient informed consent is required).^12-15^ For example, an analysis of the Connect MM Registry that enrolled 3011 patients with newly diagnosed multiple myeloma (MM) from over 200 primarily community-based sites across the US showed that 40% of the enrolled patients would likely not have qualified for MM clinical trials due to not meeting stringent eligibility criteria. These patients were more likely than RCT-eligible patients to have comorbidities and advanced disease, with poorer prognoses and outcomes.^7^ These differences lend more credence to the generalizability of results published from patient registries while highlighting the need for more inclusive and diverse populations in cancer clinical trials.^4^

Patient registries are designed to longitudinally observe cancer survival, treatment patterns, safety, clinical care, and patient-reported outcomes (PROs)^16^ in larger patient populations and for longer periods of time than clinical trials. In particular, oncology clinical trials are usually designed to follow patients through a single line of therapy until disease progression or death. Conversely, the larger patient sample sizes and longer follow-up through multiple lines of therapy in an oncology registry maximize the ability to detect safety signals and inform the safety profile of approved therapies and new compounds as they enter the market. Registries involve “primary data collection” like clinical trials and, therefore, are held to similar compliance and safety requirements as interventional trials. However, to relieve burden on the sites participating in noninterventional, observational research, registries may limit study monitoring and limit the collection of AEs to serious AEs (SAEs) only to be reported in an expedited manner, and possibly a limited number of nonserious AEs of interest. The expedited reporting of SAEs in an industry-sponsored registry likely ensures better capture of SAEs during the duration of the study than what commercial RWD providers can extract from electronic health records (EHRs) or insurance claims databases (henceforth referred to as “secondary data collection studies”). For these reasons, patient-level safety data collected from registries are often included in regulatory required, safety reporting documents, such as Risk Management Plans and Periodic Safety Update Reports, and may be included in product labeling.^17^

An additional advantage to patient registries is the ability to gather PROs prospectively over long periods of time. The integration of health-related quality of life (QoL) patient questionnaires in advanced cancer settings has allowed for a better understanding of the patient experience during treatment in the real-world setting.^8,18-20^ The administration of QoL questionnaires is a collaborative, multidisciplinary approach that, depending on the method of administration, can couple the site-level research staff with the questionnaire instrument. Most of the registries referenced in this report used paper QoL questionnaires administered to patients each time they came into the clinic for an office visit throughout their study follow-up. Administration of the questionnaires directly to the patient improves QoL completion rates, because cancer patients tend to be followed very closely for extended periods of time. However, QoL completion rates may decline as the disease progresses.^21^ Electronically administered QoL questionnaires are a more recent option available to patients and can reduce burden on sites but may present challenges to certain subsets of patients, like the elderly. The knowledge gained from QoL data collection in registries is highly regarded by the medical community and industry in the authors’ experiences (eg, high acceptance rates at key congresses) and can help inform future clinical trials.

Longer follow-up and larger size of oncology patient registries also afford a greater likelihood of capturing clinical outcomes of interest in those smaller subgroups of higher risk or sicker patients, such as the elderly,^22,23^ higher risk disease stage,^24^ poor performance status, those with impaired hepatic or renal function,^25^ or those with cytogenetics associated with poor prognosis.^26^ Longer follow-up permits researchers to study the natural history of disease in patients with cancer over time as new treatments emerge and impact the course of disease and extend survival. This information can inform treatment guidelines.^27^ For example, the National LymphoCare Study was a registry of more than 2700 patients with follicular lymphoma who were followed for approximately 8 years.^28^ A pivotal analysis from this registry demonstrated that patients who relapsed within 2 years of diagnosis had significantly poorer outcomes compared with those who progressed later.^29^ These findings inspired a similar analysis using data from the Connect Chronic Lymphocytic Leukemia Registry and similarly demonstrated that poorer outcomes were associated with earlier disease progression.^30^ Large oncology registries have informed the medical community on diagnostic patterns,^13,31-35^ treatment patterns,^36-40^ and clinical outcomes.^27,28,41^ Furthermore, by not requiring planned or scheduled clinic visits or a mandated treatment protocol, registries tend to show more heterogeneity in treatment sequencing compared with clinical trials and offer the potential to perform healthcare resource utilization analyses of patient care.^42^

It is well established that RWD generated by oncology patient registries informs clinical trial design, confirms clinical trial results, and generates hypotheses that may lead to new indications and more clinical benefits. Table 1 provides specific examples of industry-sponsored cancer registries from which data have potentially impacted patient care by either supporting a new indication or being accepted into National Comprehensive Cancer Network (NCCN) Guidelines. However, there are also many examples that may not have been referenced in treatment guidelines but were published in widely circulated, high-tier medical journals. For example, the BRiTE (Bevacizumab Regimens: Investigation of Treatment Effects and Safety) and ARIES (Avastin Registry—Investigation of Effectiveness and Safety) colorectal cancer registries provided data to support a hypothesis that sustained suppression of vascular endothelial growth factor beyond disease progression by bevacizumab results in improved survival outcomes in metastatic colorectal cancer, which informed the phase III ML18147 trial that later confirmed the registries’ findings.^43-46^ In another example, longitudinal data collected from the Connect MM Registry showed that in patients with newly diagnosed MM (NDMM), any maintenance treatment, such as lenalidomide and bortezomib, led to significantly longer median progression-free survival and overall survival compared with no maintenance without an increase in healthcare resource utilization or decrease in the patients’ QoL.^18,53^ These outcomes had not been previously reported in a meta-analysis of 3 RCTs that led to lenalidomide maintenance approval for use in patients with NDMM.^54^ More recently, the Connect MM Registry, which is still active with over 13 years of follow-up, provided data for KarMMa-RW, a comparator cohort for the single arm, KarMMa trial.^47^ Similarly, the LymphoCare Registry has generated data that have been included numerous times in NCCN guidelines and published in high-tier medical journals. For example, the LymphoCare Registry observed better outcomes in patients with follicular lymphoma (FL) who received rituximab maintenance.^55^ LymphoCare also generated key data suggesting that a proportion of patients with FL may benefit from watchful waiting.^12^ In another example, the AVIDA registry confirmed that dosing schedules and routes of azacitidine administered in patients with myelodysplastic syndromes were similar to those administered in clinical trials and, more importantly, that the route of administration did not affect outcomes.^56^ These findings helped lead to the development of an oral formulation of azacytidine.

**Table 1.** Examples of key data from Industry-Sponsored Oncology Registries that have impacted clinical practice.

Registry name	Disease indication	Sponsor	Years active	*N*	Key data	Impact
BRiTE and ARIES Bevacizumab Registries	First-line bevacizumab-treated mCRC	Genentech, Inc.	BRiTE: 2004-2008 ARIES: 2006-2012	BRiTE: 1953 ARIES: 1550	Hypothesis-generating data from these registries suggested that improved clinical outcomes are associated with utilizing bevacizumab beyond first disease progression in mCRC	Results from the 2 registries were published prior to the conclusion of the registrational, phase III ML18147 trial,^43-46^ which provided randomized data and largely confirmed these observational findings. FDA approved this indication in 2013
Connect^®^ MM	Newly diagnosed MM	Bristol Myers Squibb	2009-ongoing (through Dec 2024)	3011	Real-world data from triple-class exposed RRMM patients in North America and Europe were obtained from three types of data sources: clinical sites, the Connect^®^ MM Registry,^13^ and external research databases. The data were merged into a single data model and compared with the pivotal KarMMa study of ide-cel^47^	Data supported ide-cel approval by the FDA
National LymphoCare Study	Recently diagnosed FL	Genentech, Inc.	2004-2014	2740	An analysis of patients with FL with stages II-IV disease showed that there were no differences in OS between watchful waiting and chemoimmunotherapy or rituximab monotherapy^12^	Published in NCCN Guidelines^®^: B-Cell Lymphomas.v2.2023^48^ Informed treatment approach in adults with FL
National LymphoCare Study	Recently diagnosed FL	Genentech, Inc.	2004-2014	2740	Patients with FL who experienced disease progression within 24 months after therapy with R-CHOP had a 5-year OS of only 50% compared with 90% for patients who progressed after 24 months^29^	Published in NCCN Guidelines: B-Cell Lymphomas.v2.2023^48^ Demonstrated utility of POD24 as a predictor of survival in FL patients
National LymphoCare Study	Recently diagnosed FL	Genentech, Inc.	2004-2014	2740	Examined OS and PFS by FLIPI risk category. Demonstrated that FLIPI1 scoring criteria, which was developed in the pre-rituximab era, retained its prognostic significance in the chemoimmunotherapy era^49^	Published in NCCN Guidelines: B-Cell Lymphomas.v2.2023^48^ Supported continued use of FLIPI1 scoring criteria in the chemoimmunotherapy era
PROCEED	Sipuleucel-T treated advanced prostate cancer	Dendreon	2011-2017	1976	A prospective registry of patients with mCRPC that showed safety and tolerability of sipuleucel-T were consistent with previous findings^41^	Published in NCCN Guidelines: Prostate Cancer.v1.2023^50^ Supported approval of sipuleucel-T, a new class of cancer immunotherapeutic agents, for patients with minimally symptomatic or asymptomatic mCRPC
LORHAN	Head and neck carcinoma	MedNet Solutions	2005-2010	4243	Examined treatment patterns among patients with head and neck cancer in the community and academic settings^39^	Published in NCCN Guidelines: Head and Neck Cancers.v1.2023^51^ Supported use of intensity-modulated radiation therapy as a replacement for conventional radiation techniques
GIDEON	Unresectable hepatocellular carcinoma	Bayer HealthCare Pharmaceuticals	2008-2015	3202	Subgroup analysis of OS by liver function class demonstrated lower median OS among patients with Child-Pugh Class B liver function^25^	Published in NCCN Guidelines: Hepatocellular Carcinoma.v2.2023^52^ Together with other studies, this study formed the basis for excluding patients with poor liver function from clinical trials

Abbreviations: CLL: chronic lymphocytic leukemia; FDA: Food and Drug Administration; FL: follicular lymphoma; FLIPI: Follicular Lymphoma International Prognostic Index; HER2: human epidermal growth factor receptor 2; ide-cel: idecabtagene vicleucel; mCRC: metastatic colorectal cancer; mCRPC: castration-resistant prostate cancer; MM: multiple myeloma; NCCN: National Comprehensive, Cancer Network; OS: overall survival; PFS: progression-free survival; POD24: progression of disease within 24 months; R-CHOP: rituximab, cyclophosphamide, doxorubicin, vincristine, and prednisone; RRMM: relapsed/refractory MM.

### Value of Oncology Patient Registries in Fostering Scientific Collaboration

Registries promote collaboration between industry, the medical community, and patients in oncology research. They facilitate understanding of disease management, patient experiences, disparities in treatment, and resource utilization. Registries generally foster widespread collaboration with study sites because they typically engage more community-based sites (eg, an industry-sponsored registry may be 80%-90% community based) as interventional clinical trials are typically conducted at academic centers. In this way, registries may legitimize the clinical value of RWE by expanding beyond the clinical trial experience. Participation, in a registry study, can also provide a unique opportunity for community sites not involved in clinical trials to display or enhance their capabilities as research sites and for future clinical trials.

RWE is of particular interest to clinicians to gain information on aggregate safety and effectiveness data of approved drugs or specific treatment regimens administered within the context of typical medical practice.^8,14^ In the process, clinicians have an opportunity to communicate and provide value with colleagues sharing similar interests in registry data, to foster scientific and research collaborations within community practices, and to better understand patient perspectives pertaining to treatment choices and outcomes in a dedicated fashion.^3^

**Figure 1.**
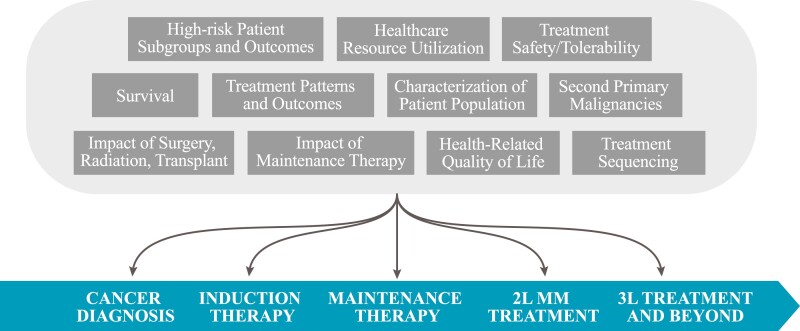
Example of published topics using data from a cancer registry by phase of treatment. Abbreviations: 2L: second line; 3L: third line.

To ensure impartiality in analysis reporting from industry-sponsored oncology registries, these studies are usually governed by an external Steering Committee (SC) composed of medical experts in the disease area and oncologists participating in the registry and may also include nurses, advanced practice providers, pharmacists, statisticians, epidemiologists, QoL experts, and patient advocates. The SC provides guidance and consultation throughout the study duration on study design, conduct and ethics, analyses, interpretation of results, and publications. This close collaboration between external SC and Sponsor as demonstrated by co-authorship on all publications is the primary key to success of industry-sponsored registries. The larger, longer nature of oncology registries enables the SC to propose a large number of longitudinal analyses to address critical medical questions (Fig. 1).

### Challenges of Industry-Sponsored Oncology Patient Registries

Oncology-focused pharmaceutical companies see the value of RWD generated by patient registries to confirm safety, clinical benefit, and value of a product. However, these studies are important commitments for the company and participating sites—they have high budgets and are resource intensive, requiring large internal and external teams (eg, contract research organizations, CROs) to conduct the study, review and analyze data, and publish findings. They also come under a lot of scrutiny by external stakeholders because they are industry sponsored. Observational studies, including registries, are not conducted with the same rigor as clinical trials, and, as a result, data quality is often questioned. Nonuniform data collection among sites can lead to missing or erroneous data. In addition, a patient’s follow-up is often at the clinician’s discretion; hence, clinic visits are not scheduled at regular intervals or mandated per protocol. Other limitations that could potentially introduce bias in analyses include channeling bias (because patients are not randomized to treatment), missed diagnostic tests and procedures, subjective disease response assessments (because clinicians are not required to follow any formal response criteria, eg, Response Evaluation Criteria in Solid Tumors [RECIST]), and limited safety data collection (eg, reporting of AEs is often limited per protocol, and there are risks of underreporting, which may be inversely related to the seriousness and severity of the AE).

With these challenges, why should pharmaceutical companies conduct registries? Industry-sponsored patient registries have an important advantage over many other types of RWD studies. First, the collaborations with key stakeholders that registries afford demonstrate to the medical community a company’s commitment to finding safe and effective treatments for patients. Second, registries provide a reliable source of longitudinal data, prospectively collected, that the company can use to address critical questions that emerge over time as the treatment and disease landscapes change. The large number of publications that come from a well conducted, well-managed registry offsets the high costs and resource commitments when compared to the single question, fee-for-service projects typical of EHR, insurance claims, or chart review data providers.

Third, with regard to data quality, primary data collection studies like registries can send queries to sites for missing and erroneous data, a feature that is generally not permitted for secondary data collection studies. Furthermore, company-sponsored registries are beholden to strict company and/or CRO processes requiring complete, accurate safety reporting for protocol-specified AEs for the company’s therapeutic products to the health authorities. The ability to query and the strict safety reporting requirements can improve the completeness and accuracy of the data. In some cases, the sponsor may also work to improve data quality by deploying remote and on-site monitoring mechanisms to conduct sample source data verification, implementing data cleaning procedures, and resolving data issues. Such examples of procedures include incorporating automated data checks in the electronic data capture system and conducting ongoing manual data review, providing formal data review guidelines to sites, conducting periodic site trainings via webinars and on-site visits, and continually tracking data to proactively identify problems with data collection.^4^ However, querying sites for more information can be burdensome for site staff, and under-resourced sites that receive too many queries may withdraw from the registry. Therefore, finding the right balance between the highest data quality and continued site participation is crucial to the ultimate success of the study.

Industry could improve the perception of industry-sponsored registries and their value in general in several ways. First, industry stakeholders should collaborate and share data, protocols, and case report forms with other industry and medical stakeholders. This is critical because oncology therapeutics are becoming more targeted for smaller patient populations. Oftentimes, one registry does not have adequate sample size to accurately evaluate effectiveness and safety of a targeted therapy. Another area for improvement is to find ways to report data to patients who participate in registries. Because patients are de-identified to the sponsor, direct access to patients is not possible. Currently, pharmaceutical companies rely on sites to provide information on the registry’s progress and publications to the patients, but staff are frequently busy and do not remember to disseminate the information the sponsor provides to the site. Public-facing registry websites and patient advocacy organizations are vehicles to indirectly report data to patients.

## Conclusion

Oncology patient registries are a valuable way to generate RWE for healthcare stakeholders for numerous reasons, including (1) they involve prospective, primary data collection; (2) they can characterize smaller patient populations typically not included in clinical trials; (3) they enable us to learn more about the course of a disease, as it changes rapidly with the rapidly changing oncology treatment landscape; (4) they allow for longitudinally collected PROs; (5) they can generate data quickly in response to urgent treatment safety and effectiveness questions; (6) they generate new hypotheses; and (7) they inform clinical trial design.

These studies are an important source of RWE that pharmaceutical companies should continue to sponsor. They cannot be replaced by secondary data collection studies. However, the success of these long-term studies depends on the medical community’s perception of their value and their continued participation.

## Data Availability

Bristol Myers Squibb’s policy on data sharing may be found at https://www.bms.com/researchers-and-partners/clinical-trials-and-research/disclosure-commitment.html.
